# Diagnostic Challenge and Therapeutic Approaches in Human Sepsis Based on the Appearance of Endotoxemia and Beta-d-Glucanemia

**DOI:** 10.3390/ijms222312900

**Published:** 2021-11-29

**Authors:** Hiroshi Tamura, Yoshiyuki Adachi

**Affiliations:** 1 LPS Consulting Office, Tokyo 160-0023, Japan; htamura@lpsct.com; 2 Department of Host Defense and Biochemical Research, Graduate School of Medicine, Juntendo University, Tokyo 113-8421, Japan; 3 Laboratory for Immunopharmacology of Microbial Products, School of Pharmacy, Tokyo University of Pharmacy and Life Sciences, Hachioji, Tokyo 192-0392, Japan

Circulating endotoxin, also called lipopolysaccharide (LPS) and (1→3)-β-d-Glucan (β-d-glucan), major constituents of bacterial and fungal cell walls, respectively, are determined as biomarkers for Gram-negative sepsis and invasive fungal diseases. These components are known to play an important role in innate immunity responses through Toll-like receptors (TLRs) and C-type lectin receptors (CLRs) to invading pathogens by sensing microorganisms [1]. In this Special Issue entitled: “Diagnostic Challenge and Therapeutic Approaches in Human Sepsis Based on the Appearance of Endotoxemia and Beta-d-Glucanemia” of the *International Journal of Molecular Sciences*, we have collected a review and original articles wherein the authors address these topics. There are 11 exciting papers, including five on endotoxins and six on beta-d-glucan. Endotoxin-related topics include review articles about antimicrobial peptides [2,3], structural diversity and molecular modification of lipid A [4], a novel marker for septic inflammation [5], and clinical interventions using an endotoxin-removal device [6]. 

First, Nagaoka et al. [2] revealed that human host defense cathelicidin peptide LL-37 exhibits multiple protective actions on septic mice by caecal ligation and puncture (CLP). Attention was also focused on the fact that such antimicrobial peptides are a new class of antibiotics not prone to bacterial resistance. The cationic peptide also induces the modulation of cell death pathways such as pyroptosis and NETosis, and the stimulation of the release of antimicrobial NETs and ectosomes in addition to its own bactericidal and LPS-neutralizing activities. Thus, we expect that LL-37 may become a potential therapeutic target for sepsis because of its unique and fundamental role in host defense. Nguyen et al. [3] reviewed the evidence for the association between skin barrier disruption in patients with atopic dermatitis and antimicrobial peptides (AMPs) such as LL-37 and human β-defensins. It has been recognized that skin and skin structures are among the most frequent sites of human bacterial infection. Thus, atopic dermatitis is frequently complicated by severe systemic inflammation in sepsis and septic shock [7]. The findings can contribute to developing new medicine to reduce the risk of severe systemic infections, because AMPs increase the levels of tight junction-related proteins and promote epidermal barrier function.

Kawahara [4] focused not only on the structural characteristics of lipid A, but also on the construction of novel lipid A through the modification of the fatty acid profile. It should be noted that he introduced an exciting and creative approach based on genetic engineering for lipid A modification. New compounds generated from remarkable achievements are expected to provide potential vaccine adjuvants and lipid A antagonistic drugs for sepsis and septic shock. Su et al. [5] reviewed the molecular bases of transglutaminases and their functional properties. Interestingly, they obtained fascinating data on the critical role of transglutaminase 2 (TG2) in an experimental model of sepsis. The immunofluorescence technique using TG2 KO mice is considered a convincing and promising approach in clarifying the involvement of TG2 in the development of sepsis. It can also be suggested that TG2 is a novel target for the diagnosis, prognosis, and effective treatment of septic disease. 

Shoji et al. [6] provided a review article on the therapeutic effect of direct hemoperfusion with a polymyxin B immobilized fiber column (PMX-DHP). This is a significant and helpful article to fully understand the history and recent progress in endotoxin removal therapy with PMX-DHP in conjunction with endotoxin detection in blood [8]. Additionally, the paper discusses a fascinating topic on the potential application for COVID-19 patients and the expected clinical indication for sepsis.

In the part on (1→3)-β-d-glucan, three review articles and three original papers are contributed, all of which present the latest and valuable findings and opinions. β-glucan is an important target molecule in the serodiagnosis of invasive fungal infections, and β-glucan promotes immune function by acting on innate immune receptors, which can also lead to immune-related diseases.

Yoshida [9] clearly shows the relationship between the diagnosis of invasive fungal infections, which are a clinical problem, and antimicrobial treatment strategies. In the review, Yoshida introduced the US, European, and Japanese guidelines on invasive fungal infections and febrile neutropenia, and explained the relationship between diagnosis and treatment clearly, showing flow chart algorithms. The importance of serodiagnosis was also discussed, especially beta-glucan detection, in empiric antimicrobial therapy and pre-emptive aggressive therapy in the treatment strategy of invasive fungal infections.

The Limulus amebocyte lysate (LAL) assay, which utilizes the (1→3)-β-d-glucan reactivity of horseshoe crab G-factor, is widely used for the detection of (1→3)-β-d-glucan in patient sera, but it is often problematic because of its cross-reactivity with (1→3)-β-d-glucan other than fungi, resulting in false positives. Kanno et al. [10] analyzed the possibility that (1→3)-β-d-glucan latent in pollen may be the cause of these false positive results, and reported that contamination of Japanese cedar pollen with G-factor reactive (1→3)-β-d-glucan induces high values of the Limulus method. Until now, (1→4)-(1→3)-β-d-glucan eluted from medical materials containing cellulose fibers was suspected to be the cause of false positive results, but pollen in the environment may also have to be considered as a false positive causative agent.

Yamanaka et al. [11] attempted to create a mutant of (1→6)-β-d-glucanase in order to obtain a protein that specifically binds to (1→6)-β-d-glucan, and succeeded in creating a detection probe with high affinity. In addition, we constructed a smart detection system as a luciferase fragment–enzyme complex that can easily evaluate the (1→6)-β-d-glucan binding activity. Since the β-glucans in the fungal cell walls of Candida and Aspergillus, which are the two major causative agents of deep-seated fungal infections, contain not only (1→3)-β-d-glucan but also (1→6)-β-d-glucan, this method is attracting attention as a detection system for fungal polysaccharides other than (1→3)-β-d-glucan. 

The LAL is a protein reagent produced from the blood cell lysate of horseshoe crabs. It is a diagnostic reagent that depends on marine wildlife resources, and from the viewpoint of biological conservation, it is desirable to switch to an alternative. The problem of false positives is also an issue that needs to be resolved. Adachi et al. [12] developed a single-molecule fluorescence detection system (SSMC) with low false positives by combining recombinant (1→3)-β-d-glucan binding protein and (1→6)-β-d-glucan binding protein produced in E. coli. The SSMC method is comparable to the sensitivity of the Limulus method and is expected to have practical application, as it solves the false positive problem. The future development of this method is noteworthy.

β-glucan is present in the cell wall of fungi and not only activates the innate immune response during infection, but also influences the development of various immune-related diseases. Dectin-1, which acts as a β-glucan receptor in humans and mice, and β-glucan binding proteins from invertebrates have been shown to interact with the characteristic higher-order structure of (1→3)-β-d-glucan in a stepwise manner in three dimensions. Chemical analysis of the interaction between β-glucan and receptor molecules is important for a deeper understanding of the molecular mechanism, but has not been investigated in detail.

Manabe and Yamaguchi [13] provide new insights into this issue and provide valuable hints for a molecular explanation of the process from ligand–receptor binding to cell activation signal generation. These findings are expected to provide important clues for the development of β-glucan assay techniques that cover a wide range of structural properties, as well as for the development and monitoring of new therapeutic agents for invasive fungal infections that lead to sepsis.

Desamero and Kakuta et al. [14] comprehensively summarized many of the results of their studies using β-glucan receptor gene knockout mice, and provided a clear picture of the progress and achievements in this research field. In particular, the genes, molecular structures, expressing cells, and signaling mechanisms of the major β-glucan receptor, dectin-1, are described in detail. The book contains an exhaustive list of studies on the investigation of fungal infection models using dectin-1 gene-deficient mice, which is valuable information for the complete β-glucan researcher. It is expected that further studies using β-glucan receptor-deficient mice will provide more and more important insights.

The editorial board wishes to thank all contributing authors for the effort and time spent on each manuscript. The Editor-in-Chief would also like to thank all the editors for their time spent reviewing, assigning and commenting on the manuscripts. The editorial team hopes that this Special Issue will be useful for future research in Gram-negative sepsis and invasive fungal diseases. Hopefully, many researchers will refer to these articles to improve the diagnosis and treatment of bacterial and fungal infections with professional success.
